# High Dose “HDR-Like” Prostate SBRT: PSA 10-Year Results From a Mature, Multi-Institutional Clinical Trial

**DOI:** 10.3389/fonc.2022.935310

**Published:** 2022-07-29

**Authors:** Donald B. Fuller, Tami Crabtree, Brent L. Kane, Clinton A. Medbery, Robert Pfeffer, James R. Gray, Anuj Peddada, Trevor J. Royce, Ronald C. Chen

**Affiliations:** ^1^CyberKnife Centers of San Diego, San Diego, CA, United States; ^2^Advance Research Associates, Santa Clara, CA, United States; ^3^Community Cancer Center, Clovis, CA, United States; ^4^Southwest Radiation Oncology, Oklahoma City, OK, United States; ^5^Benefis Sletten Cancer Institute, Great Falls, MT, United States; ^6^Sarah Cannon Research Institute, Nashville, TN, United States; ^7^Penrose-St. Francis Health Services, Colorado Springs, CO, United States; ^8^University of North Carolina at Chapel Hill, Chapel Hill, NC, United States; ^9^University of Kansas, Kansas City, KS, United States

**Keywords:** prostate cancer, SBRT, PSA nadir, HDR, relapse free rate, CyberKnife

## Abstract

**Purpose/Objective(s):**

Although ample intermediate-term prostate stereotactic body radiotherapy (SBRT) outcomes have been reported, 10-year results remain relatively sparse.

**Materials/Methods:**

Eighteen institutions enrolled 259 low- and intermediate-risk patients. Median follow-up is 5.5 years, with 66 patients followed ≥ 10 years. This SBRT regimen specifically emulated an existing HDR brachytherapy dose schedule and isodose morphology, prescribed to 38 Gy/4 fractions, delivered daily by robotic SBRT, mandating > 150% dose escalation in the peripheral zone. Androgen deprivation therapy was not allowed, and a hydrogel spacer was not available at that time.

**Results:**

Median pre-SBRT PSA 5.12 ng/mL decreased to 0.1 ng/mL by 3.5 years, with further decrease to a nadir of < 0.1 ng/mL by 7 years, maintained through 10 years. Ten-year freedom from biochemical recurrence measured 100% for low-risk, 84.3% for favorable intermediate risk (FIR), and 68.4% for unfavorable intermediate (UIR) cases. Multivariable analysis revealed that the UIR group bifurcated into two distinct prognostic subgroups. Those so classified by having Gleason score 4 + 3 and/or clinical stage T2 (versus T1b/T1c) had a significantly poorer 10 year freedom from biochemical recurrence rate, 54.8% if either or both factors were present, while UIR patients without these specific factors had a 94.4% 10-year freedom from biochemical recurrence rate. The cumulative incidence of grade 2 GU toxicity modestly increased over time – 16.3% at 5 years increased to 19.2% at 10 years-- while the incidence of grade 3+ GU and GI toxicity remained low and stable to 10 years - 2.6% and 0%, respectively. The grade 2 GI toxicity incidence also remained low and stable to 10 years – 4.1% with no further events after year 5.

**Conclusion:**

This HDR-like SBRT regimen prescribing 38 Gy/4 fractions but delivering much higher intraprostatic doses on a daily basis is safe and effective. This treatment achieves a median PSA nadir of <0.1 ng/mL and provides high long-term disease control rates without ADT except for a subgroup of unfavorable intermediate-risk patients.

## Introduction

Stereotactic body radiotherapy (SBRT) is by now a well-recognized treatment option for patients with clinically localized prostate cancer. The recognition of a low alpha-beta ratio (1), suggesting prostate cancer to be relatively more responsive to higher doses per fraction, as well as improved treatment delivery precision that limits collateral organ at risk dose exposure, is the basis for the use of hypofractionated radiotherapy for this disease, culminating in the adoption of ≤ 5 fraction regimens, delivered by SBRT methodology.

Preceding contemporary SBRT and also in parallel, a different method of extremely hypofractionated radiotherapy has used high dose rate (HDR) brachytherapy to deliver the entire course in a similar number of fractions, with excellent disease control and acceptable toxicity (2). The power and convenience of HDR brachytherapy come at the expense of requiring an invasive procedure, a requisite period of hospitalization to accomplish, and is hindered by a small set of physicians skilled at this technique nationwide.

SBRT can be designed to deliver dose fractionation and isodose morphology substantially identical to that of HDR brachytherapy, with the obvious advantages of being noninvasive and potentially more widely available (3). In spite of the potential advantages of prostate SBRT, one of the factors limiting its further use is a continued relative scarceness of long-term data – a necessary essential to document both efficacy and safety relative to other radiotherapy modalities that have a longer history.

Herein, we present the mature results of a multi-institutional clinical trial of “HDR-like” SBRT, reporting the late PSA kinetics, disease-free survival, and toxicity of this regimen to 10 years. A well-established radiation dosing schedule of 38 Gy in four fractions has demonstrated excellent efficacy with high-dose-rate (HDR) brachytherapy, and is recognized by the American Brachytherapy Society as a standard treatment option (4). The current prospective multi-center Phase II trial was designed to emulate this regimen with SBRT; both the dose fractionation and the internal prostatic isodose morphology, described in greater detail in our original study of this technique (3), while eliminating the invasiveness and inconvenience of brachytherapy.

## Material (Patients) and Methods

### Patients and Treatment

Eligible patients included those with low- and intermediate-risk prostate cancer using the D’Amico classification (5). All pathology was centrally reviewed (Bostwick Labs). Patients were treated from December 2007 through February 2012 at 18 institutions, the majority of which are community-based practices (**Appendix Table 1**). This clinical trial was registered with clinicaltrials.gov (NCT00643617) with all participating institutions receiving IRB-approval.

**Figure 1:**
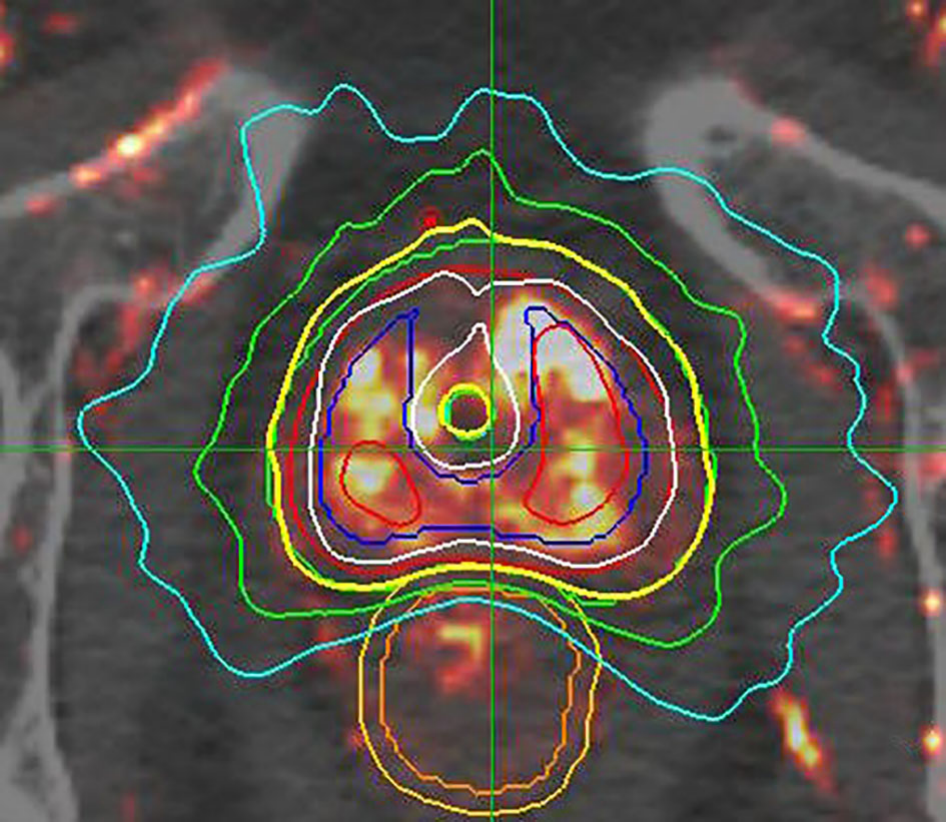
Sample HDR-like treatment plan, with contours and isodose display: This image set consists of a DCE enhanced MRI, superimposed over a standard planning CT image. The prostate GTV is contoured in red. The asymmetrically expanded PTV is contoured in green, revealing a 2 mm GTV to PTV expansion on the right (lesser involved side); a 5 mm GTV to PTV expansion on the left side (heavier involvement and with Gleason 7 disease); with manual “shaving” of GTV to PTV expansion down to zero mm adjacent to the rectum. This plan was constructed before the advent of SpaceOAR. This case is prescribed to 3800cGy/4 fractions, displayed by the yellow isodose line, with extreme conformality around the underlying green PTV contour. Additional isodose information: 125% = white, 150% = red, 75% = green, and 50% = aqua. NOTE that the yellow prescription isodose line touches, but does not breach, the outer rectal wall, and also has a central dip to relatively spare the urethra, while the 75% green isodose line touches but does not breach the rectal mucosa, defined as a 3 mm contraction from the outer rectal wall. This design morphology concentrates the greatest dose in the peripheral zone of the most heavily involved left lobe, with wider coverage margins adjacent to that region.

Patients received 38 Gy in four daily fractions of 9.5 Gy per fraction, using a fiducial-guided robotic SBRT technique (CyberKnife^®^; Accuray, Sunnyvale, CA, USA). Androgen deprivation therapy was not allowed in this trial. CT-based simulation was done with Foley catheter for urethra delineation; prostate MRI with image co-registration to the CT was encouraged but not required. The clinical target volume (CTV) included the prostate for all patients; intermediate-risk patients also included 1 cm of proximal seminal vesicles. PTV margin was a 2-mm volume expansion in all directions from the prostate, except posteriorly where the prostate abutted the rectum, which was manually adjusted on the computer to a 0-mm margin. For Gleason 7 cancer patients, the ipsilateral side(s) of the involved prostate had a 5-mm PTV expansion to cover potential extracapsular extension more thoroughly. Treatment planning coverage and normal tissue constraints are detailed in **Appendix Table 2**. The trial required plans with >1% of the PTV receiving at least 150% of the prescription dose (≥57 Gy), to emulate HDR brachytherapy dosimetry (**Figure 1**). It should be noted that this trial did not mandate a prostate size cut-off (the largest prostate volume was 155 cc). Of note, this entire trial happened before the availability of hydrogel spacers.

### Outcomes Assessed

Patients were evaluated 3, 6, and 12 months out, semiannually during years 2 through 5 and annually after that to year 10. Biochemical recurrence was defined using the Phoenix definition (nadir plus 2 ng/mL) (6). We report freedom from biochemical recurrence and clinical recurrence, stratified by risk groups. Toxicity was assessed using the Common Terminology Criteria for Adverse Events (CTCAE) version 3.0.

### Statistical Methods

The Kaplan-Meier method was used to estimate toxicity over time and freedom from recurrence. The log-rank test was used to compare risk groups. Univariate and multivariable Cox proportional hazard models were used to evaluate factors associated with freedom from biochemical recurrence. All statistical analysis was performed using SAS version 9.4 (Cary, NC), and two-sided p-values of <0.05 were considered statistically significant.

## Results

Overall, 259 patients were enrolled, with median age being 68.7 years (**Table 1**); 43% (112) were low-risk and 57% were intermediate-risk (101/147 favorable; 46/147 unfavorable). The median follow-up is 5.5 years, with 66/259 (25%) followed ≥ 10 years.

**Table 1:** Multivariable analysis for freedom from biochemical recurrence.

	Univariate		Multivariable	
	Hazard Ratio	P-value	Hazard Ratio	P-value
Age	1.084 (per year)	0.0326	1.059	0.1982
iPSA (REF: 0-4.0 ng/mL)		0.1915		0.0958
4.01-10 ng/mL 10.01-20 ng/mL	2.062 5.272		1.426 9.359	
Risk Group (REF: Low/fav int)		0.0007		0.3804
Unfavorable Intermediate	4.969		1.810	
Gleason (REF: 3 + 3)		0.0008		0.0477
3+4 4+3	3.656 10.804		2.699 8.827	
T-Stage (REF: T1c) T2a/T2b	2.939	0.0231	3.143	0.0359
# (+) Biopsy Cores	1.064 (per core)	0.5607	1.028 (per core)	0.8249

Low-risk group has no events.

T2b only has three subjects so was combined with T2a.

### PSA Response and Freedom From Recurrence

**Figure 2:**
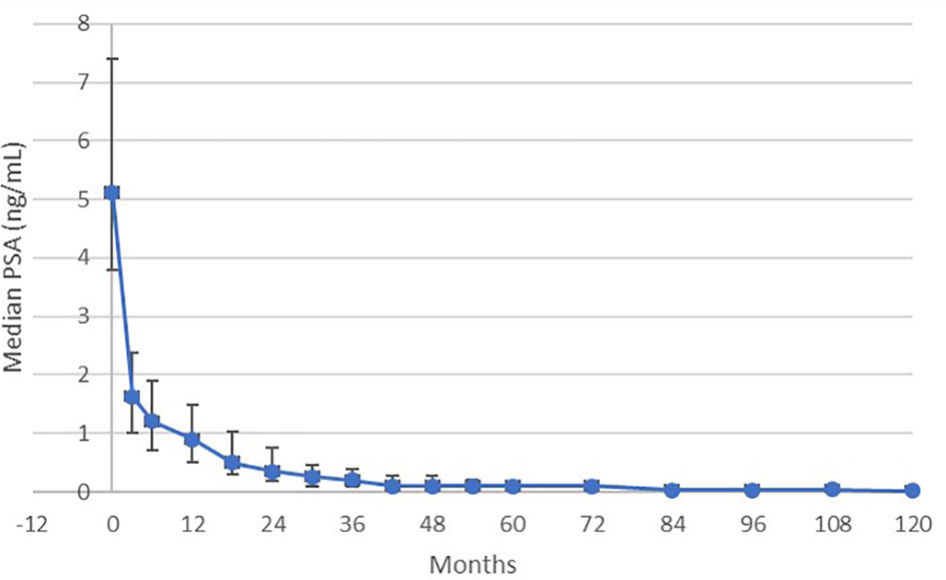
Median PSA level (with +/- one standard deviation bar) for patients on trial.

**Figure 3:**
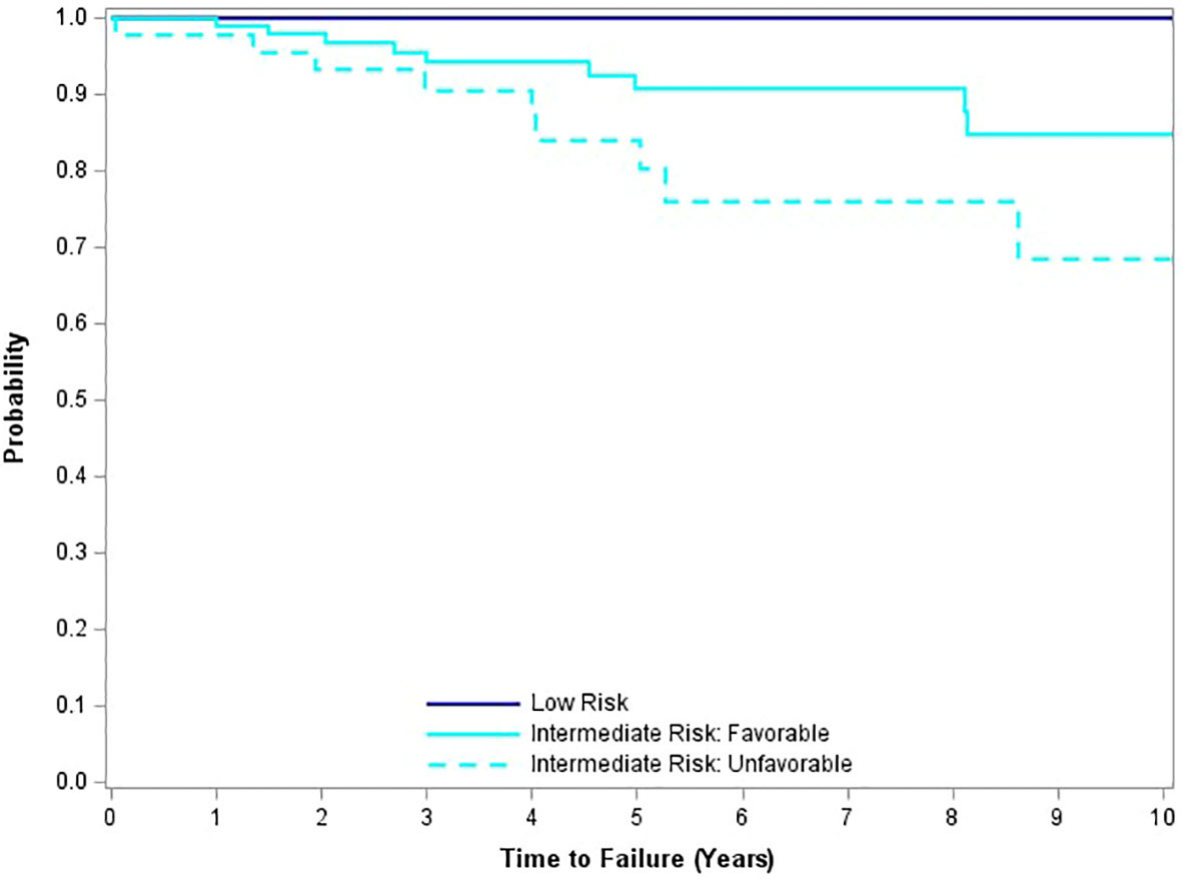
Freedom from biochemical recurrence by risk group.

From an initial median PSA level of 5.12 ng/mL, the median PSA continued to decrease to 0.1 ng/mL by 42 months, and then to < 0.1 ng/mL at year 7, maintained through year 10 (**Figure 2**). The 10-year freedom from biochemical recurrence (FFBR) measured 100% for low-risk, 84.3% for favorable intermediate-risk, and 68.4% for unfavorable intermediate-risk (p=0.0001 – univariate analysis; **Figure 3**).

For the minority of patients with clinical relapse, the pattern is primarily distant, representing 78% of the total. 10-year local relapse-free survival measured 99%, while 10-year distant relapse-free survival measured 95.5%. All clinical failures occurred in the intermediate-risk cohort, with the majority of distant failures occurring in the unfavorable intermediate-risk group. There was one prostate cancer-specific death within the first 10 years of follow-up, translating to 99.5% 10-year disease-specific survival.

On multivariable analysis, Gleason score 4 + 3 = 7 (p=0.0477) and the presence of palpable as opposed to impalpable disease (stage T2a/T2b versus stage T1c) (p = 0.0359) were significantly associated with FFBR (**Table 1**).

**Figure 4:**
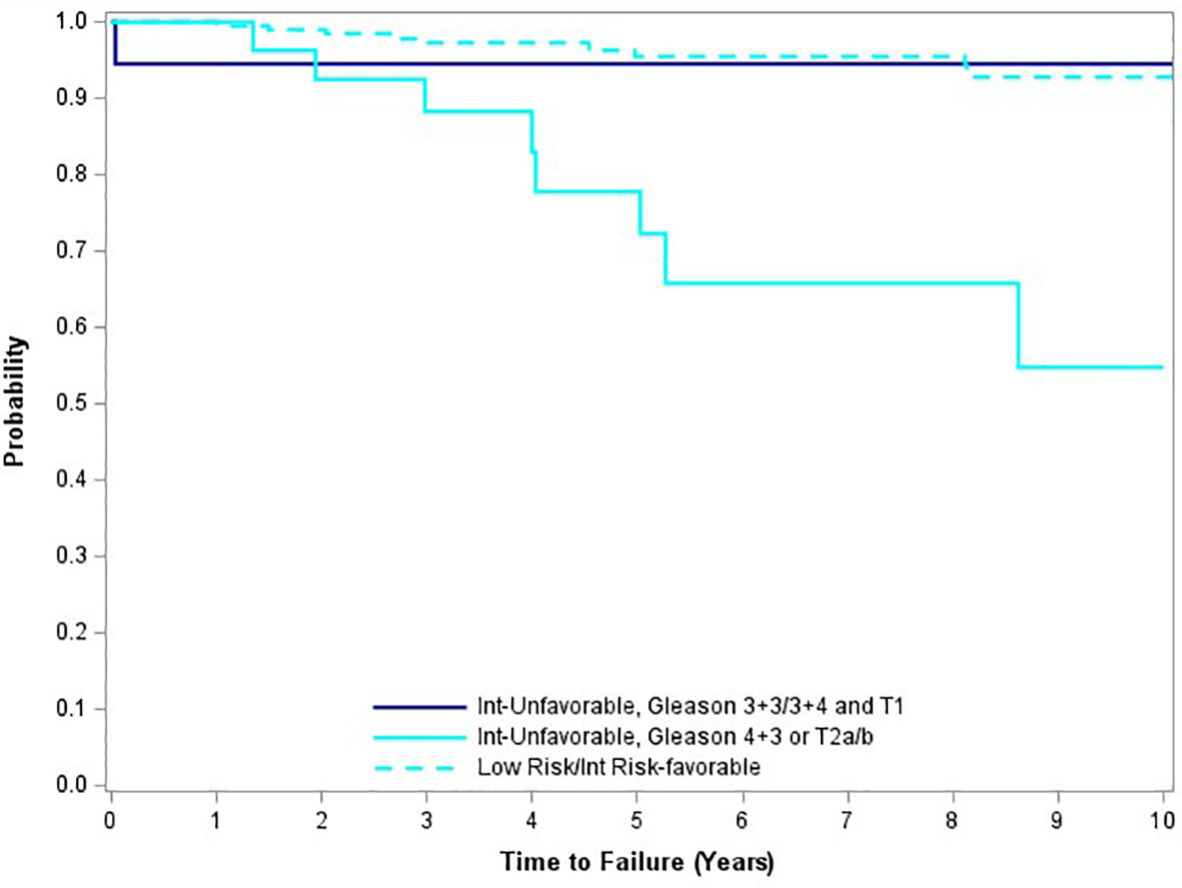
Freedom from biochemical recurrence for subgroups of unfavorable intermediate-risk patients.

Patients with Gleason score 4+3=7 and/or stage T2a/T2b disease had a 54.8% 10-year FFBF, versus 92.8% for all other patients in the trial. For the subgroup of UIR patients without a Gleason score of 4 + 3 or palpable disease, the 10-year freedom from biochemical recurrence rate was 94.4%. Although risk group classification was highly significant on univariate analysis (p=0.0001; worsened outcome with increased risk group), this finding disappeared on multivariable analysis. **Figure 4** illustrates the large curve separation of Gleason 4 + 3 = 7 and/or palpation stage T2 cases, versus all remaining study cases.

### Toxicity

Acute genitourinary (GU) toxicity (≤ 90 days) measured 35.1% for grade 2 and 1.1% for grade 3; including one patient (0.4%) with catheter dependent urinary retention and two (0.8%) with severe frequency/dysuria. Acute gastrointestinal (GI) toxicity measured 6.9% grade 2 with no grade 3 or higher acute GI events. Regarding chronic toxicity (> 3 months), the cumulative incidence of grade 2 or higher GU toxicity measured 16.3% by year 5, modestly increased to 19.2% by year 10. The cumulative incidence of grade 3 and higher GU toxicity was 2.6% at 5- and 10-years. The cumulative incidence of grade 2 GI toxicity was 4.1% at 5 and 10 years, with 1.1% so classified due to rectal bleeding. We observed no grade 3 or higher long-term GI toxicity in this study.

## Discussion

Prostate cancer radiotherapy continues to evolve, progressed from “standard” or “conventional” fractionation (8-9.5 weeks) to moderate hypofractionation (4-5.5 weeks), to an increasing prevalence of SBRT (1-2 weeks). Initially, commonly published SBRT regimens often applied 35-36.25 Gy in five fractions over 1 to 2 weeks (7–9). As there were minimal efficacy and safety data for SBRT for any fractionation at protocol inception, our dosing schedule was derived from a well-established “safe and effective” HDR brachytherapy regimen (2). Using HDR-like heterogeneous planning and a higher total dose (38 Gy in four fractions), as reported in this study, represents a more intensive treatment regimen than other prostate SBRT regimens. Philosophically, we sought to recapitulate this HDR brachytherapy dose fractionation and isodose morphology regimen as exactly as possible, using robotic SBRT as the delivery mechanism (3).

Assuming an alpha/beta ratio of prostate cancer to be 2.0, this regimen delivered an equivalent dose at 2 Gy per fraction (EQD2) of 109 Gy to the margin of the PTV, with a substantially higher dose throughout the substance of the extra-urethral prostate due to “HDR-like” isodose morphology. This equates to an (Equivalent Uniform Dose) EUD to the entire PTV of approximately 48 Gy/4 fractions (125% of the prescription isodose level), which translates to an “average” intraprostatic EQD2 of 181 Gy. This compares to an EQD2 of 83 Gy at the periphery and approximately 111 Gy “average” intraprostatic EQD2 for the common 36.25 Gy/5-fraction SBRT regimen (assumes the dose prescribed to the 83% isodose line). Compared to standard fractionation IMRT, prescribed to 80 Gy/40 fractions, both SBRT regimens are hotter, as this IMRT regimen creates an EQD2 of 80 Gy at the margin of the PTV and an “average” intraprostatic EQD2 of approximately 86 Gy (assumes the dose prescribed to the 95% isodose line, with less intraprostatic dose heterogeneity). It is worth noting that our SBRT prescription regimen is similar in concept to “micro-boosting” that is now more commonly studied in IMRT and SBRT trials. Our HDR-like SBRT regimen in essence boosted bilateral extra-urethral prostate to much higher doses than the prescription dose, and in this manuscript, we demonstrate the long-term efficacy and safety of this technique.

We show that this treatment achieves a median nadir of < 0.1 ng/mL at 7 years, continuously maintained thereafter through 10 years. This level of “surgical” PSA result is not commonly seen with prostate IMRT without ADT. Clinically, the 10-year local relapse-free survival rate measures 99%, with no additional local relapses seen after year three. Additionally, the 100% 10-year rate of freedom from any form of relapse, including biochemical relapse, in low-risk patients, is a result that has not been previously reported, to the knowledge of the authors. These attributes appear to validate the high radiobiologic potency implied by the above-described EQD2 discussion.

Although all published SBRT regimens create a low PSA nadir, the more conservatively dosed regimens (33.5 – 37.5 Gy/5 fractions) do not reach a full ablation level, with reported nadirs from 0.2 – 0.48 ng/mL (10–12). One institution published two separate post-SBRT PSA kinetic response papers, using a dose of 35-37.5 ng/mL, demonstrating that the median PSA level decreased from 0.3 ng/mL at 3 years to 0.2 ng/mL at 5.6 years of median follow-up, though only 40% of the patients in the longer-term study achieved an “ablation” PSA nadir level (11, 12). Once again, this suggests that the more conservatively dosed regimens are potent, but not routinely ablative. The more conservatively dosed prostate SBRT regimens create a PSA nadir that resembles that of dose escalated IMRT (6, 10).

Of note, a radiologically ablative regimen does not necessarily translate to cured prostate cancer either, as reported in a pooled multi-institutional SBRT dose response analysis (13). This report indicated that although 38 Gy/4 fractions produced the steepest PSA decline slope and lowest absolute PSA nadir versus all other evaluated prostate SBRT regimens, this attribute did not translate to an improved biochemical relapse-free rate versus the slightly less radiobiologically aggressive 40 Gy/5 fraction regimen. In our series, this appears particularly so for the subset of patients with unfavorable intermediate-risk disease, so classified due to Gleason score 4 + 3 = 7 and/or palpable (T2a/T2b) disease, who have a relapse rate in excess of 40% in spite of the locally ablative nature of their primary treatment.

Clinically, unfavorable intermediate-risk patients, and particularly those with the specific findings of Gleason 4 + 3 or palpation stage T2 (as opposed to T1) disease, have a higher propensity to distant relapse. As such, these patients should be more thoroughly staged prior to treatment, ideally now including a contemporary “prostate specific” PET/CT scan, which may be more sensitive to the detection of small metastatic foci that may evade conventional imaging evaluation. Additional treatment intensification measures should also be considered for these patients, potentially including the addition of prophylactic pelvic lymph node radiotherapy, the addition of androgen deprivation therapy, or both.

Interestingly, unfavorable intermediate-risk patients without the specific negative attributes described in the paragraph above had a much more favorable outcome, similar to the remainder of the patients in this series, a bifurcation in the UIR risk group that has not been previously reported to the knowledge of these authors. Possibly, this difference suggests that UIR patients with Gleason score 4 + 3 and/or palpable disease have a significantly higher metastatic potential or existing micro-metastatic disease at diagnosis, while the remainder of the UIR group could tend to have higher volume local disease of lesser metastatic potential, thus well treated by locally ablative sole modality SBRT.

In parallel, we now observe a similar UIR outcome dichotomy predicted by advanced tumor genomic profiling, specifically, the CCR score, with a result below 2.114 similarly predicting a much more favorable 10-year UIR radiotherapy efficacy result, regardless of whether or not ADT is added to the regimen (14). In the future, it would be interesting to see if our own dichotomous UIR outcome based on traditional factors has identified substantially the same prognostic bifurcation, now detected by contemporary genomic profiling.

Due to potential toxicity of a high EQD2 at the PTV margin, this trial was designed with small CTV to PTV margins (2-5 mm, with any 5 mm expansion limited to high extracapsular extension risk sub-regions). Posteriorly, there was further “shaving” to a zero mm margin, to spare the rectum. This did not lead to an excess incidence of missing extra-prostatic disease, as no posterior marginal relapses were observed. However, the result of this specific approach may not be generalizable to all SBRT methods; there remains a lack of data on the efficacy of lower dose prostate SBRT using non-CyberKnife treatment machines, with a CTV to PTV margin expansion this small.

The higher biologically effective dose delivered with “HDR-like” SBRT could also result in increased toxicity (bowel, bladder, and/or urethra). A prior study using SEER-Medicare claims data suggested high toxicity rates after SBRT, highlighting this concern (15). Although we did observe a modest further increase in the cumulative incidence grade 2 GU toxicity between year 5 (16.3%) and year 10 (19.2%), there was no further increase in grade 2 GI toxicity after year 5 (4.1%). This modest increase in urinary symptoms over time could be due to treatment, but could also be due to aging of enrolled patients progressing from a median 68.7 years at enrollment to 78.7 years of age, by 10 years later. The cumulative grade 2 GU and GI toxicity rates we report are slightly lower versus a recently published modern, aggressive conventional fractionation radiotherapy series, the “FLAME” series, which reported cumulative grade 2+ GU and GI toxicity rates of 27.1% and 10.2% in their dose-escalated arm, respectively (16). Likewise, our delayed grade 2 GU and GI toxicity incidence is virtually identical to the 10-year incidence with a well-established and more mature 81 Gy/45 fraction IMRT regimen, reported by Zelefsky, et al. – 20% and 5%, respectively, in their series versus 19.2% and 4.1% in our current report (17).

Finally and reassuringly, the low rate of grade 3 or higher GU and GI toxicity has no further progression after year 5 in our series. It is notable that we demonstrate these results delivering SBRT daily (not every other day as commonly used in other SBRT regimens). Once again, our 10-year cumulative grade 3 toxicity incidence remains competitive with modern conventional prostate IMRT - 2.6% for grade 3+ GU and 0% for grade 3+ GI toxicity through year 10 in our own series - virtually identical versus the 3% GU and 1% GI incidence reported in the Zelefsky IMRT series described above (17). Thus, we have confirmed no delayed severe toxicity surprises with this regimen.

The toxicity profile of this SBRT regimen also compares favorably with brachytherapy. Post-brachytherapy catheter dependent urinary retention has been reported following both permanent source and HDR prostate brachytherapy, with an incidence of 9% or greater (18, 19). The presently described SBRT trial had a <1% incidence of catheter dependent urinary retention. Avoidance of needle trauma, a possible contributor to acute post-brachytherapy urinary retention, might explain the low rate of retention observed in this trial.

As the 10-year local control rate in this series is 99%, perhaps there is also some room to de-escalate the total dose. In fact, subsequent to the inception of this protocol, we launched a lower dose “HDR like” prostate SBRT dose regimen – 34 Gy/5 fractions, using proportionately identical isodose morphology but scaled to the lower total dose. This regimen has very similar freedom from biochemical recurrence rates to 5 years, with a minimally higher PSA nadir value (0.1 ng/mL versus < 0.1 ng/mL). There is less confirmation of long-term efficacy with this lower dose regimen, due to its later inception, with a resulting smaller percentage of patients at risk for 10 or more years (20).

In our own practice, the practical application of the subtle differential PSA nadir and follow-up discrepancy between regimens is a tendency to apply the higher dose regimen to patients with a greater than 20-year life expectancy and/or with higher volume local disease. We more commonly use the lower dose regimen for those who have lower-volume lesions, lesser potential longevity, and/or a higher toxicity risk (e.g., large prostate, high IPSS score, prior TURP); also for those who are extremely concerned regarding potentially different quality of life implications of the different regimens.

## Conclusion

In summary, an HDR-like SBRT regimen prescribing 38 Gy/4 fractions but delivering much higher intraprostatic doses on a daily basis is safe and effective. This treatment achieves a median PSA nadir of <0.1 ng/mL and provides high long-term disease control rates without ADT except for a subgroup of unfavorable intermediate-risk patients.

## Data Availability Statement

The original contributions presented in the study are included in the article/**Supplementary Material**. Further inquiries can be directed to the corresponding authors.

## Ethics Statement

The studies involving human participants were reviewed and approved by Scripps IRB. The patients/participants provided their written informed consent to participate in this study.

## Author Contributions

All authors contributed to the data collection, the manuscript was primarily prepared by DF and RC, statistical analysis was performed by TC. All authors read and approved the final manuscript.

## Conflict of Interest

Author TC was employed by Advance Research Associates. The authors declare that this study received funding from Accuray, Inc. The funder had the following involvement in the study: Assistance with data collection and statistical support.

The remaining authors declare that the research was conducted in the absence of any commercial or financial relationships that could be construed as a potential conflict of interest.

## Publisher’s Note

All claims expressed in this article are solely those of the authors and do not necessarily represent those of their affiliated organizations, or those of the publisher, the editors and the reviewers. Any product that may be evaluated in this article, or claim that may be made by its manufacturer, is not guaranteed or endorsed by the publisher.
