# Supplementary material for: High Dose “HDR-Like” Prostate SBRT: PSA 10-Year Results From a Mature, Multi-Institutional Clinical Trial
Source: Front Oncol. 2022 Jul 29;12:935310. doi: 10.3389/fonc.2022.935310 (PMC9373838; doi:10.3389/fonc.2022.935310)
Supplement: Supplementary file 1 [file Table_1.docx]

**APPENDIX Table 1: List of participating institutions**

| *Site name* | *State* |
| --- | --- |
| AtlantiCare Cancer Care Institute | NJ |
| Elmhurst Memorial Hospital | IL |
| East Texas Medical Center Cancer Institute | TX |
| California Cancer Center | CA |
| JFK Medical Center | FL |
| Nevada Cancer Research Foundation | NV |
| Long Beach Memorial Medical Center | CA |
| University Of South Alabama Mitchell Cancer Institute | AL |
| Oakwood Hospital and Medical Center | MI |
| Albert C. Mak, M.D., Inc | CA |
| Penrose-St. Francis Health Services | CO |
| Genesis Healthcare Partners | CA |
| Sarah Cannon Research Institute | TN |
| Benefis Sletten Cancer Institute | MT |
| Southwest Radiation Oncology | OK |
| Cyberknife of Brandon | FL |
| CyberKnife Center of Tampa Bay | FL |
| University of North Carolina at Chapel Hill | NC |
