# Supplementary material for: High Dose “HDR-Like” Prostate SBRT: PSA 10-Year Results From a Mature, Multi-Institutional Clinical Trial
Source: Front Oncol. 2022 Jul 29;12:935310. doi: 10.3389/fonc.2022.935310 (PMC9373838; doi:10.3389/fonc.2022.935310)
Supplement: Supplementary file 2 [file Table_2.docx]

**APPENDIX Table 2: Dose constraints specified in the protocol**

| **Organ** | **Dose Constraint** |  |  |  |  | | |  |  |  |  |  |
| --- | --- | --- | --- | --- | --- | --- | --- | --- | --- | --- | --- | --- |
| Planning Target Volume (PTV) | 38 Gy delivered in 4 fractions of 9.5 Gy per fraction.  95% PTV encompassed within prescription isodose volume. | | | | | | | | | | | |
|  | PTV volume receiving at least 150% prescription dose (57 Gy) will be >1% | | | | | | | | |  |  |  |
| Rectum outer wall | Maximum dose 100% prescribed dose (38 Gy) | | | |  | | |  |  |  |  |  |
|  | Minor variation: Maximum dose 100.1-110% prescribed dose (41.8 Gy) | | | | | | | |  |  |  |  |
|  | Major variation: Maximum dose >110% prescribed dose (41.8 Gy) | | | | | | | |  |  |  |  |
| Rectal mucosa | Maximum dose 75% prescribed dose (28.5 Gy) | | | |  | | |  |  |  |  |  |
|  | Minor variation: Maximum dose 75-90% prescribed dose (34.2 Gy) | | | | | | | |  |  |  |  |
|  | Major variation: Maximum dose >90% prescribed dose (34.2 Gy) | | | | | | | |  |  |  |  |
| Bladder | Maximum dose 120% prescribed dose (45.6 Gy) | | | |  | | |  |  |  |  |  |
|  | Highest 10% (D10) ≤ 110% prescribed dose (41.8 Gy) | | | | |  | |  |  |  |  |  |
| Urethra | Maximum dose 120% prescribed dose (45.6 Gy) | | | |  | | |  |  |  |  |  |
|  | Highest 10% (D10) ≤ 110% prescribed dose (41.8 Gy) | | | | |  | |  |  |  |  |  |
|  | Highest 50% (D50) ≤ 105% prescribed dose (39.9 Gy) | | | | | |  |  |  |  |  |  |
| Conformality index for normal tissues | Ratio of prescription isodose volume and PTV will be ≥ 1.0 and ≤ 1.5  Minor Variation: ≤ 0.99 or ≥ 1.51-2.0 | | | | | | | |  |  |  |  |
